# *Heidelberg Risk Sport-Specific Stress Test*: A Paradigm to Investigate the Risk Sport-Specific Psycho-Physiological Arousal

**DOI:** 10.3389/fpsyg.2019.02249

**Published:** 2019-10-18

**Authors:** Marie Ottilie Frenkel, Sylvain Laborde, Jan Rummel, Laura Giessing, Christian Kasperk, Henning Plessner, Robin-Bastian Heck, Jana Strahler

**Affiliations:** ^1^Institute for Sport and Sport Sciences, Heidelberg University, Heidelberg, Germany; ^2^Institute of Psychology, German Sport University Cologne, Cologne, Germany; ^3^Psychological Institute, Heidelberg University, Heidelberg, Germany; ^4^Steroid Laboratory, Department of Internal Medicine I and Clinical Chemistry, University Hospital Heidelberg, Heidelberg, Germany; ^5^Department of Psychology and Sport Science, Justus Liebig University Giessen, Giessen, Germany

**Keywords:** anxiety, cortisol, heart rate variability, high risk sports, psychological and physical demands in sports

## Abstract

In risk sports with medium to high risks of injury (e.g., surfing, free solo climbing, wingsuit flying), athletes frequently find themselves in unexpected and threatening situations. Elevated psycho-physiological stress responses to these situations might have tremendous consequences for their performance as well as for their long-term health. To gain a better understanding of the psycho-physiological response to such events, innovative, externally valid and standardized stress induction protocols are needed. Therefore, the aim of this paper is to introduce and evaluate a risk sport-specific stress protocol, i.e., the *Heidelberg Risk Sport-Specific Stress Test* (HRSST), which utilizes fear of falling as the stressful event. Climbing novices were asked to climb up a 12 m high wall. Then, participants were requested to “jump into the rope”, leading to a secured fall of about 3 m. This imposed physical danger assumed to elicit psycho-physiological responses. Self-reported state anxiety, salivary cortisol, and heart rate/heart rate variability were measured before, during, and after the HRSST. Results of four independent studies that investigated the psycho-physiological response to the HRSST in 214 participants were analyzed, leading to conclusions about the stressor’s effectiveness. Results showed that self-reported state anxiety consistently increased after the HRSST in all four experiments (moderate to large effects). The results of the physiological indicators were inconclusive. Salivary cortisol significantly increased after the HRSST in one of four experiments (small effect sizes). Although heart rate significantly increased during the “jump in the rope” in experiment 1, heart rate variability significantly decreased after the HRSST in only one of three experiments (small effect sizes). Findings suggest that the HRSST is a valid method to induce risk sport-specific emotional stress, but effects on physiological stress markers were rather minor. To sum up, in case of appropriate sports climbing facilities, the HRSST appears to be a cost-efficient and promising stress induction protocol: It offers the possibility to investigate risk sport-specific stress responses and their underlying mechanisms in climbing novices. These findings may also find application in professions in which individuals are exposed to risky situations, such as police officers, medical first responders, firefighters and military personnel.

## Introduction

In sports, increased psycho-physiological stress does not only have negative consequences for the athlete’s health, but can also impair sports performance (Paulus et al., 2009; Röthlin et al., 2016). Examining the mechanisms linking sport-specific (emotional) stress exposure with motor performance and the well-being of athletes is therefore an important endeavor in sport-psychological research. In risk sports, athletes are often required to execute cognitive and motor skills under demanding, potentially stressful environments, in which performance failure might result in severe injuries or even death (Brymer and Schweitzer, 2013; Arijs et al., 2017; Frenkel, 2019). While the acute stress response to these demands is highly adaptive (Sapolsky et al., 2000), frequent or chronic activation of psycho-physiological responses can result in allostatic load, i.e., “the wear and tear on the body” due to compensatory chronic stress responses (McEwen, 1998, 2013). Detrimental effects related to an increased allostatic load could be assumed for both mental and physical health, e.g., anxiety and depression (McEwen, 1998; Herman, 2013) or decreased performance (Nunes et al., 2014).

In more detail, increased environmental demands elicit a stress response if potentially threatening and perceived as succeeding the individual’s coping abilities (Lazarus and Folkman, 1984). This stress response can be differentiated into its psychological, physiological, and behavioral components. Psychological responses involve an emotional (anxiety, affect or mood, emotional stress) and a cognitive dimension (appraisal, rumination, blackout) with the latter focusing on the subjective evaluation of an individual’s ability to cope with the stressor (Lazarus and Folkman, 1984). As part of the emotional stress response, anxiety has been characterized as an aversive emotional and motivational state when facing uncertainty or a perceived existential threat (Eysenck et al., 2007). The Multidimensional Theory of Competitive State Anxiety proposes two dimensions, cognitive anxiety as the negative evaluation of performance, and somatic anxiety as a physiological dimension (Martens et al., 1990). The response to stressful and demanding events also comprises the activation of physiological systems, including the activation of the autonomic nervous system (ANS) with the release of catecholamines, and the hypothalamic-pituitary-adrenal (HPA) axis with the secretion of glucocorticoids, mainly cortisol, from the adrenal cortex (Dickerson and Kemeny, 2004). Cortisol can be reliably assessed in saliva (sCort; Hellhammer et al., 2009) and autonomic reactivity can be examined via heart rate and heart rate variability (HRV; Appelhans and Luecken, 2006). HRV represents the time interval between successive heart beats (Berntson et al., 1997). The neurovisceral integration model (Thayer et al., 2009) assumes that vagally-mediated HRV indexes self-regulation ability. Higher cardiac vagal activity, the activity of the vagus nerve reflecting cardiac functioning (Berntson et al., 1997; Laborde et al., 2017), is associated with better stress resilience and executive performance (Thayer et al., 2009). In high stress situations presenting important metabolic demands, a larger decrease in cardiac vagal activity has been suggested to be adaptive (Laborde et al., 2018). The root mean square of the successive differences (RMSSD) is one of the HRV markers reflecting cardiac vagal activity. The behavioral response finally complements this cascade of responses and ranges from flight or avoidance to the successful, active use of coping strategies.

Importantly, stress responses, particularly anxiety and increased cortisol levels, might have tremendous negative impacts on perceptual motor performance (Nieuwenhuys and Oudejans, 2012, 2017; Hermans et al., 2014). So far, only a few experimental studies in laboratory settings have investigated the consequences of stress on athletic performance (for a review see Fuchs and Gerber, 2017; Frenkel et al., 2019). In a sample of tennis players, stress induction through arithmetic exercises led to a significant negative association between the sCort reaction and tennis serve performance (Lautenbach et al., 2014). Another study of the same research group (Lautenbach, 2017) investigated the putting performance of golf players following a physical stressor (cold pressor task, CPT; Hines and Brown, 1932). In response to stress caused by having to put one’s hand into ice-cold water, sCort levels increased and attentional bias for negative sports-related words in the Stroop test decreased significantly. Noteworthy, golf performance remained unaffected.

Experimental psychoneuroendocrine stress research has proposed various models and standardized protocols to investigate acute stress reactivity under laboratory conditions. These protocols enable the assessment of stress responses with high internal validity making use of psychosocial stressors (Trier Social Stress Test; Kirschbaum et al., 1993) physical stressors (CPT; Hines and Brown, 1932), cognitive tasks (e.g., Stroop word color test; Stroop, 1935) or exercise stressors such as the bicycle ergometer test (Allgrove et al., 2008). From the multitude of published stress protocols, those involving social-evaluative and performance components seem to be the most effective ones to stimulate endocrine and autonomic reactivity (Dickerson and Kemeny, 2004). This has been confirmed in a recent within-subject design study showing physically demanding stressors particularly inducing autonomic stress responses and psychosocial stressors particularly inducing endocrine responses (Skoluda et al., 2015). As also shown in this report (Allen et al., 2017), the *Trier Social Stress Test* (TSST; Kirschbaum et al., 1993) is considered to be the most effective stress protocol in terms of robustness, and reliability of the activation of psycho-endocrine responses and the number of subjects showing a pronounced stress response. The TSST combines evaluative threat to the social self, cognitive performance, uncontrollability, and novelty to induce psychological load. While this protocol is commonly used to induce stress, it might not be feasible in some occasions. In particular, its ecological validity and transferability to real life conditions has been questioned in general population samples (Henze et al., 2017) and even more so in athlete populations (Rohleder et al., 2007). Overall, studies employing population-specific stressors have been related to even higher psycho-physiological responses, e.g., sport competitions (Kivlighan and Granger, 2006).

Interestingly, no standardized protocol employing a sport-specific stressor has been established so far (Fuchs and Gerber, 2017; Frenkel et al., 2019). Simulated job interviews or arithmetic tasks do not adequately reflect stressors that typically occur in elite or risk sports. Therefore, Fuchs and Gerber (2017) explicitly point out the necessity to develop innovative, sport-specific stress protocols with high ecological validity. Here, bicycle ergometer tests (e.g., cycling at high intensity for 8 min; Skoluda et al., 2015) have been widely used as a sport-specific stressor. However, this protocol does not elicit a strong increase in endocrine stress markers (e.g., Skoluda et al., 2015) and does not capture the psychological components of stress which are likely to have an additional impact on athletes’ performance. In risk sports with medium to high risks of injury, athletes frequently find themselves in situations that are characterized by unexpected stimuli and in which they often put their physical (and mental) health at risk.

**FIGURE 1:**
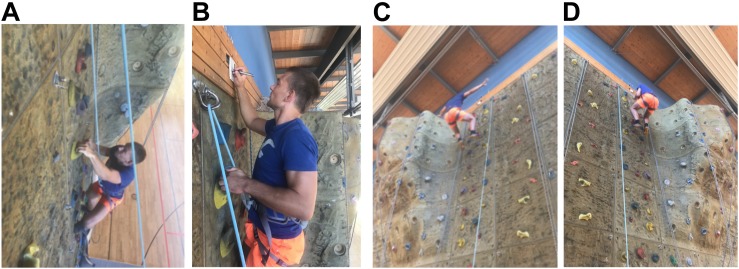
Picture of the climbing **(A)**, the assessment in the climbing wall **(B)** and the jump into the rope **(C,D)**. **(C)** Shows a jump in an exemplary manner, while in **(D)** an insecure jump, with hands on the rope, can be observed. **(A–D)** With friendly permission of the climber Thomas Stoll, the photographer Tina Völkl and the belayer Marie Ottilie Frenkel.

Following the need for ecologically valid stress protocols in sports, we developed a risk sport-specific stress test, the so-called *Heidelberg Risk Sport-Specific Stress Test* (HRSST), and investigated its application feasibility and its potential to elicit psycho-physiological stress responses. The HRSST utilizes one of the most effective psycho-physiological threat events: Fear of falling. Height and the possibility to fall present real physical danger and thereby elicit negative affective states (Draper et al., 2008, 2010; Nieuwenhuys et al., 2008). In short, subjects are asked to climb up a wall (top rope; see footnote 2 and Figure 1) and are unexpectedly asked to jump into the rope. The current paper combines data from different research projects that all employed the HRSST to answer specific research questions. For an evaluation of the psycho-physiological effects of the HRSST, we combined relevant data (psychological and psychophysical stress responses) from four independent laboratory experiments.

The aim of this paper was to present a standardized protocol for the induction of risk sport-specific stress in the laboratory and to evaluate the effectiveness of the HRSST in stimulating psycho-physiological responses. Each of the four studies was analyzed separately, but findings will be combined in an integrated discussion. Psychological responses were assessed by state anxiety. Changes in sCort and HR(V) reflected activation of the HPA axis and ANS activity, respectively.

To investigate the effectiveness of the HRSST, the following hypotheses were tested:

• Hypothesis H1: State anxiety increases in response to the HRSST.
• Hypothesis H2: sCort increases in response to the HRSST (20 min after the jump).
• Hypothesis H3: Heart rate increases in response to the HRSST (at the moment of the jump).
• Hypothesis H4: HRV (RMSSD) decreases in response to the HRSST (after the stress induction).

## Materials and Methods

### Participants

In sum 214 young men, with at least some previous sports experience (i.e., students from a high school with sports profile, students either enrolled in the degree of sport science or participating in university sports programs) aged between 16 and 41 years (*M_*age*_* = 21.99, *SD* = 2.95) were enrolled in the studies reported here. Participants did not report any current or chronic medical or psychiatric diseases (e.g., heart conditions or depression). Participants were excluded from the study when they had more than 5 h of climbing experience (*n* = 98; to maximize the effectiveness of the stress induction; Ogden, 2012), or reported a particular fear of heights (*n* = 19), consumed medication containing compounds affecting our outcome measures (*n* = 4), or had injuries or refused to participate (*n* = 44). Two participants had to be excluded from analyses because one participant did not jump and one participant consumed branched amino acids and creatine during the experiment.

In experiment 1, 30 male sport science students of the Heidelberg University aged between 20 and 32 years (*M_*age*_* = 23.47, *SD* = 3.28) took part. The sample of experiment 2 included 35 high school students of seven high schools in Heidelberg aged between 16 and 19 years (*M_*age*_* = 17.12, *SD* = 0.97), whose academic profile had a special focus on sports. In experiment 3, 88 male students of the Heidelberg University (either enrolled in the degree of sport science or in university sports programs) aged between 18 and 31 years (*M_*age*_* = 22.47, *SD* = 2.73) participated. In experiment 4, the sample consisted of 71 male sport science students or students participating in university sports programs at the Heidelberg University, aged between 19 and 41 years (*M_*age*_* = 24.90, *SD* = 4.82).

Participants volunteered to take part in the experiments. Participants of experiments 3 and 4, but not of experiments 1 and 2, were reimbursed. In all experiments, participation was voluntary and adult participants as well as parents/legal guardians of all non-adult participants provided written informed consent. The study’s design was approved by the ethical committee of the Faculty of Behavioral and Cultural Studies of Heidelberg University.

### Design and Procedure

The aim of the present report was to evaluate the psychological (anxiety) and physiological (sCort, heart rate, HRV) responses to the HRSST via pre/post-manipulation comparisons. The number of measurement points of the variables varies across the experiments due to the specific research questions addressed in these experiments (for experimental design details of experiments 1–4 see Supplement 1).

In all experiments, participants were instructed to refrain from smoking, eating, or drinking any beverages except water at least 1 h before the study and during testing. All testing sessions were conducted between 12:00 am and 8:00 pm when cortisol levels are most stable (Kudielka et al., 2004). Participants were instructed not to talk to each other about the content of the study until the end of the study. To ensure standardization, all experiments followed a written protocol that described both the test procedure as well as the instructions given by the experimenters.

Upon arrival, participants were equipped with the wireless chest strap (experiments 1 and 2) or ECG recorder (experiments 3 and 4). At the first measurement point (*t*_1_), participants reported their state anxiety and the first saliva sample was taken. After sitting quietly for a few minutes, a baseline measurement of heart rate or HRV was recorded (duration in experiments 2 and 3 was 5 min, in experiment 4 1 min). Since stress responses are maximal in response to unpredictable tasks (Dickerson and Kemeny, 2004), baseline measurements before the HRSST were conducted in a room outside the sports hall. Until the moment when entering the sports hall, participants had no knowledge about the kind of motoric task they had to accomplish. This set-up should minimize the development of any expectations concerning the upcoming climbing task.

**FIGURE 2:**
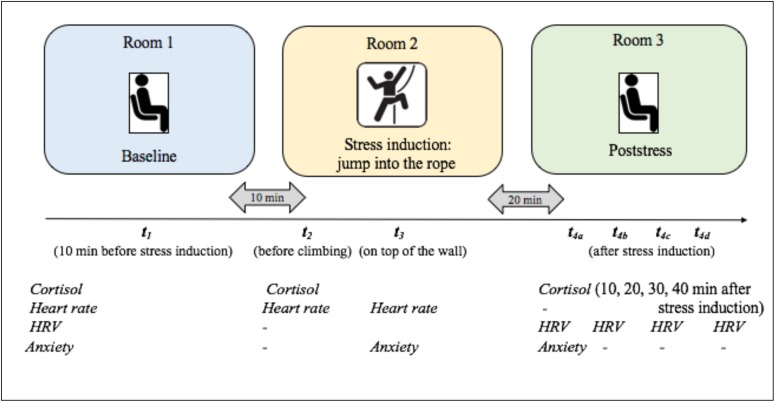
Graphical summary of the HRSST examination procedure. The study took place in three rooms. Participants underwent different physiological measurements, filled out questionnaires and completed the *HRSST*, including the climbing task. Times of measurement for the separate variables differed depending on the experiment and variables: *sCort* was measured three up to five times. *Heart rate* was measured three times. *Heart rate variability* (*HRV)* was calculated for five to six periods. *Somatic state anxiety* was surveyed three times. During all four experiments, the same labeling was used for the different times of measurement. Therefore, for example, measurement time *t*_3_ always refers to the measurement on top of the climbing wall.

Next, participants were led to the sports hall (see Figure 2) and were introduced to their belay partner.^1^ The belayer asked them to put on a harness and gave instructions for the climbing task: The participants were asked to climb to the top of the 12 m high climbing wall (*toproping*^2^; see Figure 1). In experiment 1, participants provided a saliva sample after receiving the instructions (*t*_2_). After climbing to the top of the climbing wall, participants filled out the anxiety thermometer (*t*_3_) in experiments 2–4. Then, the belayer gave further instructions for the unexpected jump into the rope: The participants were asked to “jump into the rope”^3^ and were made aware of the free fall of 2–3 m resulting from the jump. They were instructed to keep their hands off the rope, to lean backwards with the upper body in order to protect their face from potential abrasions and to land simultaneously with both feet on the wall.

If a participant displayed at least three of five previously defined abort criterions (shaking legs, slow-down and solidification of movements, cramping, loud and panting breathing or repeated asking for further instructions) before reaching the top, the HRSST was aborted and the participant was excluded from further testing and analyses (*n* = 1). In the moment of the jump, the experimenter started a digital stop watch (CASIO, HS-3V-1RET) to time the following salivary samples (*t*_4_*_*a–d*_*; see Figure 2). In experiment 1, heart rate was measured at this time. After the jump, participants were lowered by the belayer. Participants were led to the third room where the remaining measurements (measurement of state anxiety, *t*_4_*_*a*_*; saliva samples and HRV measurements, *t*_4_*_*a–d*_*) were taken. In all experiments, participants filled out different personality questionnaires not relevant for the current study purpose (see Supplement 1). Afterward, participants were thanked, compensated (in experiments 3 and 4) and fully debriefed.

### Measures

#### Self-Reported State Anxiety

In experiment 1, self-reported somatic state anxiety was assessed using the German version of the Competitive State Anxiety Inventory-2 Revised (Wettkampf-Angst-Inventar; WAI-S; Ehrlenspiel et al., 2009) at two measurement points: During baseline measurements before the HRSST (*t*_1_) and immediately after the jump (*t*_4_). The WAI-S consists of 12 items which are rated on a four-point scale ranging from 0 to 4 (“*not at all*” – “*a bit*” – “*considerably*” – “*extremely*”). The 12 items can be allocated to the subscales *somatic anxiety* (*som*), *cognitive anxiety* (*cog*), and *confidence* (*conf*). For the evaluation of the HRSST, the subscale *somatic anxiety* was chosen, since the HRSST aims to elicit somatic stress responses. An example item for the *somatic anxiety* subscale is “In the present moment… my heart throbs.” Internal consistencies for the subscale somatic anxiety were between α = 0.75 und α = 0.85, which is similar to the consistency found in the norm sample (α = 0.81; Ehrlenspiel et al., 2009).

To capture the changes in self-reported state anxiety during the HRSST in more detail, state anxiety was assessed with the one-item Anxiety Thermometer (Houtman and Bakker, 1989) in experiments 2–4. The anxiety thermometer captures the current feelings of anxiety by asking the question “How do you rate your current feelings of anxiety?” on a 10 cm visual analog scale, ranging from 0 = *no anxiety at all* to 10 = *extreme anxiety*. The anxiety thermometer was applied at three measurement points: During baseline measurements (*t*_1_), during climbing at the highest point of the climbing wall (*t*_3_) and immediately after the jump (*t*_4_). Through its high economy, the anxiety thermometer allows an easy, quick and reliable measurement of anxiety, even during climbing in the climbing wall. Houtman and Bakker (1989) report test-retest reliabilities of 0.60–0.70.

#### Salivary Cortisol

sCort was repeatedly assessed to estimate the endocrine responses to the HRSST. Participants were required to chew on a synthetic swab (Salivette, Sarstedt GmbH, Nümbrecht) for 1 min (Inder et al., 2012). Samples were taken before the HRSST (considered baseline) and up to four 10 min intervals after the jump (10, 20, 30, and 40 min after the jump). The number of measurement points varied across the experiments (see Figure 2). Since sCort levels usually peak 20 min after stressor on-set (Kirschbaum and Hellhammer, 2000), the measurement point *t*_4_, 20 min after the jump, was of central interest. At the end of the testing day, saliva samples were frozen and stored at −20°C until analyses. In experiment 1, the biochemical analyses were conducted by the laboratory “Dresden LabService GmbH,” Germany. In Experiments 2–4, samples were analyzed by the steroid laboratory of the Pharmacological Institute of the Heidelberg University Hospital, Germany. As the same assay was used, results from both institutes should be comparable. After thawing, the samples were centrifuged at 3000 rpm for 5 min which resulted in a clear supernatant of low viscosity. Salivary concentrations were determined using chemiluminescence immunoassay with high sensitivity (IBL International, Hamburg, Germany). The intra- and interassay coefficients were below 8%.

#### Heart Rate

In experiment 1, heart rate was continuously recorded using a wireless chest strap transmitter and corresponding monitor (Garmin Forerunner 305) worn on the wrist. Heart rate charts were reproduced in the Garmin software. Due to limitations in the Garmin software, punctual measurements of heart rate were used for further analyses. Baseline measurements of heart rate were recorded in a sitting position for 5 min at *t*_1_ since the lowest heart rates were identified at this time point. Heart rate at *t*_3_ was assessed in the moment of the jump, as soon as all extremities were off the climbing wall.

#### HRV

In experiments 2–4, HRV was recorded continuously by either a wireless chest strap transmitter and monitor worn on the wrist (Polar RS800) in experiment 2 or a wearable, portable, externally applied ECG recorder and wireless transmitter (eMotion Faros 180°) with two disposable ECG pre-gelled electrodes (Ambu L-00-S/25, Ambu GmbH, Bad Nauheim, Germany) in experiments 3 and 4. The negative electrode was placed in the right infraclavicular fossa (just below the right clavicle) while the positive electrode was placed on the left side of the chest, below the pectoral muscle in the left anterior axillary line. Baseline measurements of HRV were taken in a sitting position for 5 min before the HRSST. Artifact free time points of 1 min duration were chosen directly prior to the time points of saliva sampling, while participants filled out questionnaires in a sitting position. According to recent recommendations (Laborde et al., 2017), the RMSSD was calculated for quantification of cardiac vagal activity. HRV analyses were carried out with Polar Software in experiment 2 and with Kubios HRV (Biosignal Analysis and Medical Imaging Group, University of Eastern Finland, Finland) in experiments 3 and 4. A medium degree (0.25 s) for the correction of artifacts was chosen for the analysis using Kubios for the R-R signal in Experiment 2. For experiments 3 and 4, artifacts were corrected manually by analyzing the ECG signal (Laborde et al., 2017).

### Data Processing and Statistical Analyses

Initially, data of all variables were inspected for any missing or extreme values. To identify extreme values, boxplots were created separately for all measurement points. *Tukey-far-out* was chosen as a criterion for extreme values, i.e., values which were more than the triple interquartile range above/under the 75%/25% quartile were identified as extreme values (Tabachnick and Fidell, 2007). However, outliers were not excluded, since it was assumed that individual stress responses can vary within a great, still natural range. Outlier analyses served as a method to detect inconsistencies within the data of a participant.

Data was also checked for normal distribution. If the Kolmogorov-Smirnov test was found to be significant, analyses were conducted using log-transformed values following the recommendation of Kirschbaum (1991). Subsequently, only sCort values were log-transformed. To enhance comprehensibility, back transformed values are used in figures and tables.

To investigate the effect of the HRSST on anxiety, sCort, heart rate and HRV, repeated measures AN(C)OVAs were computed, depending on the number of measurement points in the respective experiment. According to the recommendation (Kirschbaum, 1991), sCort baseline level (at *t*_1_) was used as covariate. In correspondence, baseline heart rate and baseline HRV were also set as covariates. Greenhouse-Geisser corrected *p*-values were reported when the assumption of sphericity was violated as indicated by the Mauchly-Test. In case of a significant main effect of time, Bonferroni corrected paired t-tests between baseline and the other measurement points were calculated as *post hoc* tests to detect the effect of interest.

IBM SPSS Statistics 24 was used for all statistical analyses, *p* < 0.05 is considered significant and as a measure of effect size, η^2^*p* was presented.

## Results

Descriptive data of all variables can be found in Table 1.

**TABLE 1:** Descriptive statistics of salivary cortisol, heart rate, heart rate variability and anxiety in experiments 1–4.

**Variable**	**Experiment**	***M* (*SD*) at *t*_1_**	***M* (*SD*) at *t*_2_**	***M* (*SD*) at *t*_3_**	***M* (*SD*) at *t*_4a_**	***M* (*SD*) at *t*_4b_**	***M* (*SD*) at *t*_4c_**	***M* (*SD*) at *t*_4d_**
sCort	1	10.88 (6.99)	–	–	11.1 (7.18)	13.18 (7.72)	–	–
	2	6.05 (3.23)	6.26 (3.58)	–	8.85 (6.03)	–	–	–
	3	10.06 (9.19)	–	–	9.55 (6.82)	10.41 (6.86)	9.44 (5.99)	8.5 (4.81)
	4	9.14 (3.75)	–	–	8.84 (5.03)	8.88 (5.41)	7.88 (5.25)	–
Heart rate	1	70.89 (12.86)	96.64 (16.97)	131.18 (17.1)	–	–	–	–
HRV	2	57.81 (26.26)	–	–	43.7 (27.34)	35.87 (18.89)	39.69 (19.71)	42.5 (22.31)
	3	45.63 (17.48)	–	–	48.45 (20.02)	45.49 (18.8)	47.29 (17.44)	48.48 (17.08)
	4	42.76 (23)	–	–	59.58 (33.4)	43.58 (20.45)	46.71 (28.73)	46.45 (24.99)
Somatic anxiety/general anxiety	1	5.04 (1.37)	–	–	6.25 (1.8)	–	–	–
	2	1.89 (1.31)	–	2.73 (1.93)	2.21 (2.12)	–	–	–
	3	0.87 (0.85)	–	3.52 (2.45)	1.31 (1.58)	–	–	–
	4	0.9 (1.1)	–	3.34 (2.3)	1.17 (1.26)	–	–	–

**Heart rate was measured in experiment 1, HRV in experiments 2–4. Somatic anxiety was measured in experiment 1, general anxiety in experiments 2–4. M: mean; SD: standard deviation; description of the measurement points: t_1_: baseline (approximately 10 min before the stress induction), t_2_: before climbing, t_3_: on top of the climbing wall, t_4a–d_*: *after stress induction (for cortisol at 10 min intervals; for HRV at 5 min intervals).**

### Psychological Stress Responses

#### Subjective State Anxiety

**FIGURE 3:**
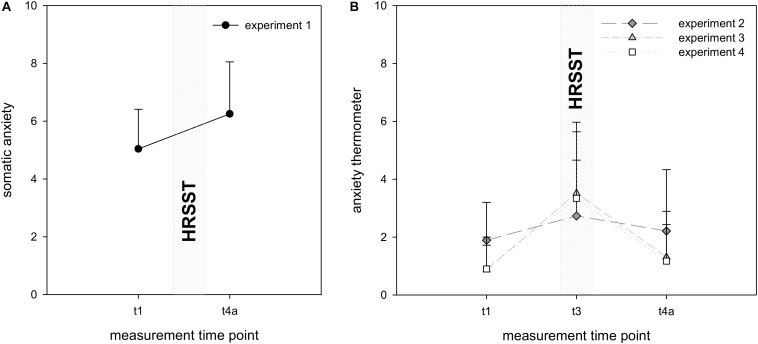
Changes in anxiety in experiment 1 **(A)** and experiments 2–4 **(B)**. Self-reported state anxiety was measured at *t*_1_ (10 min before the HRSST), at *t*_3_ (at the moment of the jump into the rope) and after the HRSST at *t*_4_*_*a*_* (10 min after the jump into the rope). Number of measurement points varies across experiments. Error bars represent standard deviations.

To evaluate the psychological response to the HRSST, somatic state anxiety (experiment 1) and general state anxiety (experiments 2–4) were analyzed. Descriptive analyses revealed an increase of anxiety in response to the HRSST in all four experiments. Levels returned to baseline until the end of the experiment (see Figure 3).

In experiment 1, the significant main effect of time in the 1 × 2 ANOVA confirmed the hypothesized increase of anxiety, *F*(1, 27) = 18.99, *p* < 0.001, η^2^*p* = 0.41. In experiments 2–4, the 1 × 3 ANOVA also revealed significant main effects of time [experiment 2: *F*(2, 66) = 3.12, *p* = 0.05, η^2^*p* = 0.09; experiment 3: *F*(2, 170) = 93.03, *p* < 0.001, η^2^*p* = 0.52; experiment 4: *F*(1.60, 280) = 86.63, *p* < 0.001, η^2^*p* = 0.55]. *Post hoc* analyses showed that anxiety significantly increased in the climbing wall as compared to the baseline in experiments 2–4 [experiment 2: *t*(34) = −3.29, *p* < 0.01; experiment 3: *t*(85) = −10.66, *p* < 0.001; experiment 4: *t*(70) = −9.61, *p* < 0.001]. In experiment 3, anxiety was still significantly higher than baseline after the HRSST at *t*_4_*_*a*_* [*t*(85) = −2.88 *p* = 0.01], whereas in experiments 2 and 4, anxiety did not significantly differ from baseline at this measurement point [experiment 2: *t*(34) = −0.96, *p* = 69; experiment 4: *t*(70) = −1.82, *p* < 0.14].

In conclusion, anxiety significantly increased in response to the HRSST in all four experiments but the timing of returning back to baseline levels differed between experiments.

### Physiological Stress Responses

**FIGURE 4:**
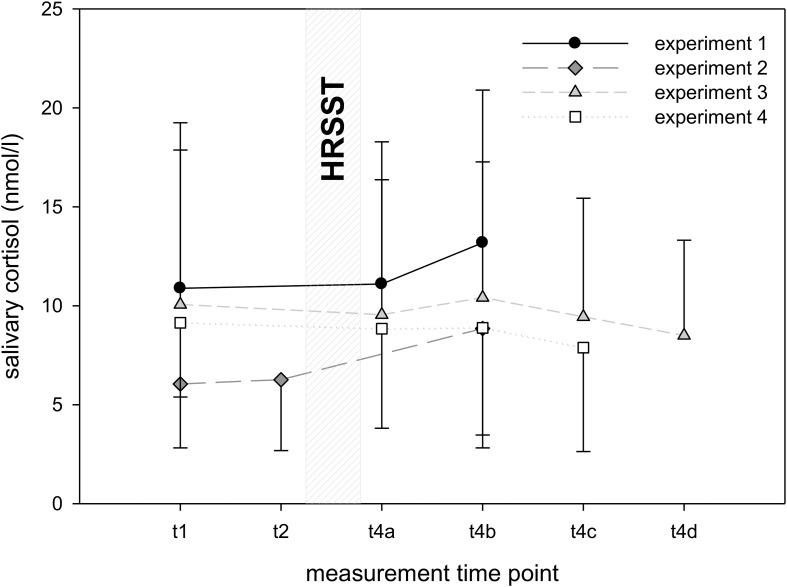
Salivary cortisol levels in experiments 1–4. Note. Salivary cortisol was measured at *t*_1_ (10 min before the HRSST), at *t*_2_ (directly before the HRSST) and after the HRSST at *t*_4_*_*a–d*_* (10, 20, 30, and 40 min after the jump into the rope). Number of measurement points varies across experiments. Error bars represent standard deviations.

To detect physiological responses to the HRSST, sCort, heart rate (experiment 1) and HRV (experiments 2–4) were analyzed. The changes in the variables within each experiment are depicted in Figure 4.

#### Salivary Cortisol

Descriptive data of sCort reveals greatest responses in experiments 1 and 2, whereas in experiments 3 and 4, sCort levels remain relatively stable (see Figure 4). Noteworthy are the high standard deviations in all experiments (between 3.23 und 7.72) and a high variation of individual averages in sCort (e.g., in experiment 1 for the baseline measurement Min = 2.6; Max = 26.6 nmol/l).

The 1 × 2 ANCOVAs (sCort baseline as covariate) detected significant main effects of time in experiment 1 [*F*(1, 26) = 8.80, *p* < 0.01, η^2^*p* = 0.25] and in experiment 2 [*F*(1, 32) = 9.90, *p* < 0.01, η^2^*p* = 0.24]. In experiment 1, *post hoc* analyses revealed that sCort neither significantly increased from baseline to 10 min after the jump [*t*(27) = −0.82, *p* = 0.84] nor from baseline to 20 min after the jump, *t*(27) = −2.09, *p* = 0.09. In experiment 2, a non-significant difference between baseline and *t*_2_ (before climbing after receiving instructions about the task) suggests that participants did not experience anticipatory stress, *t*(33) = −0.69, *p* = 0.99. However, sCort significantly increased 20 min after the jump compared to baseline, *t*(33) = −3.31, *p* < 0.01. In experiment 3 [*F*(3, 231) = 1.63, *p* = 0.18, η^2^*p* = 0.02] and experiment 4 [*F*(2, 1.62) = 0.99, *p* = 0.27, η^2^*p* = 0.02], sCort did not change significantly.

In summary, sCort was significantly higher 20 min after the HRSST compared to baseline in experiment 2, but not in the other experiments.

#### Heart Rate

**FIGURE 5:**
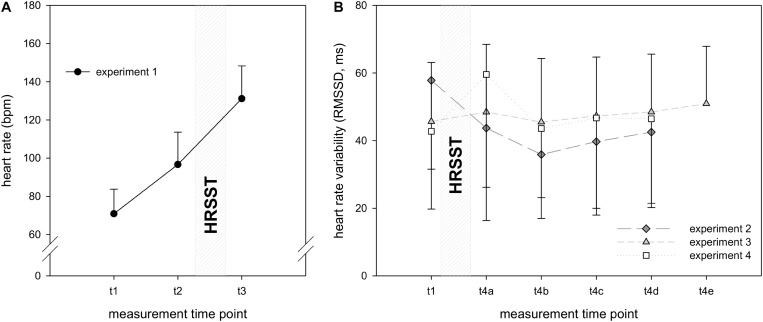
Heart rate/heart rate variability in experiments 1–4. Heart rate **(A)**/HRV **(B)** was measured at *t*_1_ (10 min before the HRSST), at *t*_2_ (before climbing), at *t*_3_ (at the moment of the jump into the rope) and after the HRSST at *t*_4_*_*a–d*_* (experiments 2 and 3: first, second, third and fourth 5 min periods after the jump into the rope; experiment 4: first, second, third and fourth 1 min period after the jump into the rope, explicitly 10, 20, 30, 40 min later). Number of measurement points varies across experiments. Error bars represent standard deviations.

Descriptive analyses showed that in experiment 1, heart rate increased in response to the HRSST, peaking at the moment of the jump into the rope (see Figure 5A). For experiment 1, the 1 × 2 ANCOVA (baseline as covariate) yielded a significant main effect of time, *F*(1, 26) = 31.75, *p* < 0.001, η^2^*p* = 0.55. *Post hoc* analyses revealed that heart rate significantly increased from baseline to the start of the climbing task [*t*(27) = −11.41, *p* < 0.001] and compared to the moment of the jump, *t*(27) = −16.35, *p* < 0.001.

#### Heart Rate Variability

Descriptive HRV data showed inconsistent patterns in experiments 2–4 with increasing RMSSD in experiment 2, stable values in experiment 3, and decreasing RMSSD in experiment 4. Noteworthy are the high standard deviations in all experiments (see Figure 5B).

In experiment 2, the 1 × 4 ANCOVA (baseline as covariate) showed a significant main effect of time, *F*(4, 336) = 3.79, *p* < 0.01, η^2^*p* = 0.04. *Post hoc* analyses demonstrated that RMSSD was significantly lower 10 min after the jump [*t*(33) = 9.48, *p* < 0.001], 20 min after the jump [*t*(33) = 6.09, *p* < 0.001], 30 min after the jump [*t*(33) = 6.08, *p* < 0.001] and 40 min after the jump [*t*(33) = 9.14, *p* < 0.001] as compared to baseline. In experiment 3 [*F*(3, 93) = 0.66, *p* = 0.58, η^2^*p* = 0.02] and experiment 4 [*F*(1.97, 131.63) = 0.49, *p* = 0.69, η^2^*p* = 0.01], RMSSD did not change significantly.

In summary, heart rate significantly increased in response to the instructions of the climbing task as well as to the instructions of the jump in comparison to baseline. In contrast, the expected decrease of RMSSD to the HRSST was only demonstrated in experiment 2, but not in experiments 3 and 4.

## Discussion

The present study proposes a new risk sport-specific stress protocol, the HRSST. The HRSST aims to induce psycho-physiological stress in laboratory settings through a climbing task with a subsequent, unexpected jump into the rope, thereby employing fear of falling as its main stress component. To evaluate the effect of the HRSST on the stress responses, psychological (i.e., anxiety) and physiological (i.e., sCort, heart rate/HRV) parameters were measured. Our first hypothesis was supported, with all four experiments confirming that self-reported anxiety increased after the stress induction with moderate (experiment 1) to large (experiment 3) effects. Our second hypothesis was partially supported by one of four experiments showing sCort increases in response to the HRSST with small effect sizes. Concerning hypothesis 3, a large effect in terms of HR reactivity was shown (of note, this parameter was only examined in experiment 1). In one of three experiments, evidence for the expected decrease in HRV with a small effect-size was provided.

In comparison to the mean increase in *self-reported state anxiety* in experiments 2–4, anxiety in experiment 1 was only slightly elevated in response to the HRSST. This finding might be explained by the timing of the measurement points and the anxiety components assessed in these particular experiments. Due to the length of the questionnaire in experiment 1, anxiety was assessed retrospectively after climbing, whereas in experiments 2–4, the one-item anxiety thermometer allowed testing in the climbing wall. Assessing state anxiety in the moment of interest is likely to generate more reliable data than retrospective testing. Additionally, the questionnaire in experiment 1 (WAI-S; Ehrlenspiel et al., 2009) assesses somatic anxiety, whereas the anxiety thermometer captures all components of anxiety. Subsequently, this finding might suggest that cognitive anxiety might be perceived as greater than somatic anxiety during the HRSST. In conclusion, the finding of increasing *anxiety* in response to the HRSST fits into the literature of risk sport/fear of falling (Draper et al., 2008, 2010; Nieuwenhuys et al., 2008; Giles et al., 2014; Baláš et al., 2017) and other physically threatening situations, e.g., in the context of the police (Landman et al., 2016; Giessing et al., 2019). In several studies, anxiety was increased in the experimental condition inducing a stronger psychological response compared to control conditions (Draper et al., 2008; Giles et al., 2014; Landman et al., 2016).

*sCort* significantly increased in response to the HRSST with a small effect size in one of four experiments. This finding is in line with the literature on other physical stressors that also include a psychological component such as the socially evaluated cold pressor test (SECPT; Schwabe et al., 2008): Adding the psychological component to the original physically stressing CPT increased the HPA axis response (Schwabe et al., 2008). The discrepancies between significant results in experiment 2 and the non-significant cortisol response in experiments 1, 3, and 4 could be influenced by random fluctuations in sample characteristics or differences in the individual study’s design. Nevertheless, the mixed pattern of results highlights the need to explore the potential active components of the stressor to further develop the paradigm and to increase its replicability. One candidate moderator of the cortisol effect might be the personality trait sensation seeking (Rosenblitt et al., 2001). In a previous study (with data from experiment 1), we found that high sensation seekers showed no changes in sCort in response to the HRSST, whereas low sensation seekers showed an average increase in sCort of 5 nmol/l (Frenkel et al., 2018). In experiment 2 reported here with a mixed sample of high and low sensation seekers, the average increase of sCort in response to the HRSST was around 2.5 nmol/l. These findings suggest that future studies using the HRSST should control for sensation seeking either statistically or in the compilation of the sample.

*Heart rate* significantly increased at the moment of the jump into the rope in experiment 1. This increase in response to the HRSST is comparable to an increase in heart rate in response to other risk sport-specific stressors (e.g., Breivik, 1999). Elite parachute jumpers, alpine and rock climbers were tested in an unknown, unexpected situation that involved perceived physical risk: They were pushed into a pool from a 5 m diving board, sitting in a white water kayak. This resulted in a significant increase in average heart rate from 100 bpm at start to 146 bpm when subjects were pushed into the pool. In experiment 1, participants showed a mean heart rate of 100 bpm after climbing with an additional mean increase of 30 bpm at the moment of the jump into the rope. Besides holding on to the climbing wall, no extra physical demands were placed on the participants in this phase of the HRSST. Therefore, the increase in heart rate might be explained by the instructions for the jump into the rope provided in this time period. Together these findings suggest that the anticipation of unexpected, threatening and potentially physically harmful tasks elicit physiological stress responses in sports samples.

However, *RMSSD* significantly decreased in only one of three experiments. According to the neurovisceral integration model (Thayer et al., 2009), a larger RMSSD withdrawal is considered adaptive when metabolic demands are high. Although participants responded to the situational demands of the HRSST with a decrease in RMSSD in experiment 2 and were not even fully recovered 30 min after the HRSST, participants in experiments 3 and 4 did not show any changes in RMSSD. However, it should also be noted that the inspection of the physiological data revealed high standard deviations for RMSSD. When interpreting the data, it should be considered that these great individual variations might result in instable sample statistics and unreliable estimations of inferential population parameters. Further, the devices used to assess HRV may have influenced the diverging results related to RMSSD. In experiment 2, in which the significant decrease in RMSSD was observed, a Polar chest belt and R-R intervals analyses were used. Potentially, the rapid movements during the fall might have created severe artifacts, that cannot be corrected as precisely with R-R interval data as with electrocardiogram data obtained in experiments 3 and 4. Consequently, the use of the Polar chest belt in the fall situation may have produced artifacts that confounded the HRV signal during the fall in experiment 2.

In summary, the evaluation of the effects shows that the HRSST has the potential to be psycho-physiologically arousing for (risk) sport samples. Further research is necessary to determine which aspects of the HRSST activate stress responses. According to previous reviews (Dickerson and Kemeny, 2004), novelty, unpredictability and physical threat in the HRSST are valid candidates. Possibly, climbing novices experience the unusual strain of certain muscle groups (i.e., forearm musculature, bizeps) as challenging. In addition, participants might have interpreted the physical activation by the climbing task as anxiety. However, since the samples in the experiments consisted of experienced and fit athletes, it was assumed that the climbing task would only elicit low levels of physical demand. Certainly, leaving solid ground and climbing into a height of 12 m elicit stress in climbing novices (Draper et al., 2008, 2010; Giles et al., 2014). In experiments 3 and 4, participants were required to spend extra time in this height while filling out a questionnaire. This left them in an instable standing position with only one hand to hold on to the climbing wall. Accordingly, participants already experienced anxiety after climbing before receiving instructions for the jump. The heart rate data suggests that the instruction and anticipation of the unpredicted task (i.e., jump into the rope) further increased stress levels. Additionally, participants were explicitly made aware of the physical threat inherent in the task by emphasizing the risk of injuries in the instructions (see section Materials and Methods). Uncertainty was further increased by the instruction “jump whenever you are ready,” which does not provide any specification of the timing of the jump. Specifying the timing of the jump (e.g., by count down) might have resulted in higher action orientation, whereas the lack of specification might have increased the state orientation which is accompanied by dysfunctional thoughts about the situation and emotional states (Kuhl, 1985).

In conclusion, the novel situation (climbing up to a height of 12 m), the unexpected task to jump into the rope and the physical threat are considered as the central, stress-inducing components of the HRSST.

### Strengths and Limitations

In medium to high risk sports, the HRSST exhibits greater ecological validity than the current established stress protocols, such as the TSST or CPT. External validity might be given for various extreme sport athletes, such as bungee jumpers, skiers and surfers, but less so for low risk sports athletes (e.g., running, swimming). The application of the HRSST is limited to climbing novices. The novelty of the situation as well as the perceived threat of the physical integrity are considered as the key elements to induce stress. Due to prior exposure to climbing and falling in height, experienced climbers are very likely not to perceive these situational demands of the HRSST as succeeding their coping abilities.

The safe and appropriate conduction of the HRSST requires significant expertise, including highly qualified staff (i.e., experienced belay partners holding a valid climbing trainer license) and appropriate climbing facilities and equipment. Nevertheless, the advantage of the HRSST is the tailored, externally valid stress induction for specialized samples that face novel, physically threatening situations with unpredictable stimuli. Standardized guidelines for the procedure in case of extremely anxious participants (i.e., specifying anxiety symptoms and abortion criteria) ensure an ethical conduction of the protocol. Besides risk sports, the HRSST might also be relevant for high-risk professions, such as military, police, rescue teams and special forces in humanitarian aid. Individuals in these professions are also required to act in novel, unpredictable, life-threatening situations, in which performance failure has dramatic consequences. Likewise, the literature on high-risk profession still lacks a standardized, externally valid, safe stress protocol comparable to the HRSST (e.g., Strahler and Ziegert, 2015; Giessing et al., 2019). Therefore, the HRSST can be considered as a starting point for the development of population-specific stress paradigms in high-risk settings.

Several limitations regarding the samples, the devices used to measure heart rate and HRV, the climbing motor task, and the experimental design need to be acknowledged. When interpreting the effects of the HRSST, it needs to be considered that only male participants were tested and generalizability to women is restricted. Future studies need to include female samples (controlling for menstrual cycle phase and hormonal contraceptive use) and samples of varying age. Age effects and sex differences in the stress responses to the HRSST are quite likely (Kirschbaum et al., 1999). Since climbing is a muscularly demanding task, future studies should examine age- and sex-specific differences in physical demands after the climbing to ensure a clear distinction between physical and psychological stress. However, analyses of the heart rate data implicated no major physical exertion in our experiments. Therefore, the potential influence of physical exertion on cortisol levels in the current report seems negligible.

So far, no study including appropriate control conditions (e.g., with traversal climbing) was conducted to evaluate the effects of the HRSST. First, it might be interesting to compare the responses to the HRSST with responses to current, established stress protocols (e.g., TSST, CPT). Second, it is necessary to implement a control condition requiring the performance of a motor task similar to the climbing task. Bouldering appears to be an appropriate option (see Nieuwenhuys et al., 2008; Englert et al., 2015). Bouldering is a form of climbing that is performed in 1–2 m height without the use of ropes or harnesses. Consequently, bouldering does not involve the height/fear of falling nor physical threat of bad injuries. Therefore, missing stress responses to bouldering would confirm the stress-inducing effects of the height and jump into the rope within the HRSST.

According to the previous literature on factors maximizing endocrine stress responses (Dickerson and Kemeny, 2004; Schwabe et al., 2008; Skoluda et al., 2015), adding a social-evaluative component to the protocol might increase the reliability of the HPA axis activation after the HRSST. Even though participants are observed by the experimenter and are recorded by camera in the present study, verbally emphasizing the evaluative and competitive nature of this test may amplify stress responses.

Furthermore, the integration of additional stress measures allows further conclusions. Additional stress biomarkers, such as alpha amylase, sex steroids or metabolic process markers, should confirm the adrenocortical results. Psychologically, it is promising to supplement the self-reports on anxiety with video analyses of facial expressions. Participants’ facial expressions can be captured via video cameras during the experimental procedures. The recordings will be analyzed with the automated facial coding software FaceReader (Noldus Information Technology, Wageningen, The Netherlands). The FaceReader is a software that enables automatic analysis of facial expressions, which can use the video data acquired from a webcam. The FaceReader has already been used extensively in previous research (Platt et al., 2013; Kunz and Lautenbacher, 2014) in order to analyze emotions based on the Facial Action Coding System (Ekman and Oster, 1979).

## Conclusion

The aim of the present report was to introduce and evaluate an innovative sport-specific stress protocol. The HRSST features a climbing task with a subsequent jump into the rope which can be considered an externally valid method to induce risk sport-specific stress. When appropriate climbing facilities and qualified staff are guaranteed, the protocol allows easy, cost-effective and safe testing. While the HRSST reliably increases reported anxiety levels, findings on the physiological stress responses are inconsistent but there is at least evidence for small-to-medium effects on the physiological level. The novelty, unpredictability as well as physical threat of the situation are considered to be stress-inducing. We hope the HRSST will become a useful method for risk sport-specific stress induction in future research.

## Data Availability Statement

The raw data supporting the conclusions of this manuscript will be made available by the authors, without undue reservation, to any qualified researcher.

## Ethics Statement

The study’s design was approved by the ethical committee of the Faculty of Behavioral and Cultural Studies of Heidelberg University.

## Author Contributions

The article at hand was mutually developed. Each author contributed to the study planning, data analysis, and interpretation with an additional focus on their respective area of competence. All authors contributed crucially in drafting the aim of the study, concretizing the design, and finishing the manuscript. MF, JR, LG, and R-BH did conduct the experiments. MF was essentially responsible for the statistical analysis, supported by LG, JR, SL, HP, CK, and JS. MF interpreted the data together with SL, JR, LG, and JS. MF wrote the first draft of the manuscript. Additionally, MF managed the communication between all authors during the development of the manuscript. All authors have examined and agreed to the submitted version of the manuscript. MF has assumed responsibility for being the corresponding author, and for keeping all co-authors informed of the progress through the editorial review process, the contents of the reviews, and any revisions made.

## Conflict of Interest

The authors declare that the research was conducted in the absence of any commercial or financial relationships that could be construed as a potential conflict of interest. The handling Editor is currently editing co-organizing a Research Topic with one of the authors SL, and confirms the absence of any other collaboration.

## Footnotes

1 The term “belayer” describes the climbing partner who stays on the ground to secure the climber through the rope.

2 “Toproping*”* is a style of climbing, in which the climber is securely attached to a rope which passes up through an anchor system at the top of the climb and down to the belayer at the foot of the climb. The belayer takes in slack rope during the climb, so that the climber only falls a short distance when loosing his/her hold.

3 “Jumping into the rope” is part of the “fall training.” Beginners experience jumping and falling often as arousing. This circumstance was used to induce psycho-physiological arousal and stress. The belayer artificially prolonged the fall by releasing 1–2 m of the rope in the moment of the jump. This is a common procedure when belaying to ensure dynamic and save fall.
