# Supplementary material for: Heidelberg Risk Sport-Specific Stress Test: A Paradigm to Investigate the Risk Sport-Specific Psycho-Physiological Arousal
Source: Front Psychol. 2019 Oct 18;10:2249. doi: 10.3389/fpsyg.2019.02249 (PMC6813738; doi:10.3389/fpsyg.2019.02249)
Supplement: Supplementary file 1 [file Table_1.doc]

**Supplement 1** Specifics of the four experiments

| Stu-dy | Subjects | Design | Paradigm | Main focus | Outcomes |
| --- | --- | --- | --- | --- | --- |
| 1 | 30 male sport science students,  20-32 yrs,  *M_age_* = 23.47,  *SD* = 3.28 | mixed-factorial design (between: HSS vs. LSS; within: repeated measurements of several variables related to stress reactivity) | HRSST | sensation seeking | somatic anxiety,  sCort,  heart frequency,  HRV |
| 2 | 35 male high school  students,  16-19 yrs,  *M_age_* = 17.12,  *SD* = 0.97 | mixed-factorial design (between: HSS vs. LSS and Depletion vs. Non-Depletion; within: repeated measurements of several variables related to stress reactivity) | HRSST + depletion task | sensation seeking, trait self-control, climbing endurance, climbing competence/ experience | state anxiety, arousal, valence,  sCort,  HRV |
| 3 | 88 male sport science students,  18-31 yrs,  *M_age_* = 22.47,  *SD* = 2.73 | mixed-factorial design (between: Depletion vs. Non-Depletion; within: repeated measurements of several variables related to stress reactivity) | HRSST + depletion task | sensation seeking, state self-control, intrinsic motivation, climbing endurance, climbing efficacy | state anxiety,  arousal, valence,  sCort,  HRV,  face reader |
| 4 | 71 male sport science students,  19-41 yrs,  *M_age_* = 24.90,  *SD* = 4.82 | two-factorial within-subject-design (innersubject-factor condition: breathing vs. film; innersubject-factor time: repeated measurements of several variables related to stress reactivity) | HRSST + breathing exercise (or control condition) | sensation seeking, state self-control, intrinsic motivation, climbing endurance | state anxiety,  arousal, valence,  sCort,  HRV,  face reader |

Note. Some of the experiments also involved an experimental manipulation, i.e. depletion paradigm, breathing technique or watching TV. This circumstance will not be considered during analyzes but our discussion will reflect on that.
